# Miocene Shark and Batoid Fauna from Nosy Makamby (Mahajanga Basin, Northwestern Madagascar)

**DOI:** 10.1371/journal.pone.0129444

**Published:** 2015-06-15

**Authors:** Tsiory H. Andrianavalona, Tolotra N. Ramihangihajason, Armand Rasoamiaramanana, David J. Ward, Jason R. Ali, Karen E. Samonds

**Affiliations:** 1 Département de Paléontologie et d’Anthropologie Biologique, Faculté des Sciences, Université d’Antananarivo 101, Antananarivo, Madagascar; 2 Department of Earth Sciences, The Natural History Museum, Cromwell Road, London, SW7 5BD United Kingdom; 3 Department of Earth Sciences, University of Hong Kong, Pokfulam Road, Hong Kong, China; 4 Department of Biological Sciences, Northern Illinois University, DeKalb, Illinois, United States of America; Raymond M. Alf Museum of Paleontology, UNITED STATES

## Abstract

Madagascar is well known for producing exceptional fossils. However, the record for selachians remains relatively poorly known. Paleontological reconnaissance on the island of Nosy Makamby, off northwest Madagascar, has produced a previously undescribed assemblage of Miocene fossils. Based on isolated teeth, ten taxonomic groups are identified: *Otodus*, *Carcharhinus*, *Galeocerdo*, *Rhizoprionodon*, *Sphyrna*, *Hemipristis*, *Squatina*, *Rostroraja*, *Himantura* and Myliobatidae. Six are newly described from Madagascar for the Cenozoic (*Galeocerdo*, *Rhizoprionodon*, *Sphyrna*, *Squatina*, *Rostroraja* and *Himantura*). In association with these specimens, remains of both invertebrates (e.g., corals, gastropods, bivalves) and vertebrates (e.g., bony fish, turtles, crocodylians, and sirenian mammals) were also recovered. The sedimentary facies are highly suggestive of a near-shore/coastal plain depositional environment. This faunal association shares similarities to contemporaneous sites reported from North America and Europe and gives a glimpse into the paleoenvironment of Madagascar’s Miocene, suggesting that this region was warm, tropical shallow-water marine.

## Introduction

Madagascar is one of the world’s highest-priority biodiversity “hotspots” with high endemism of plants and animals [1]. These groups have been greatly shaped through isolation; originally wedged between Africa and India as part of the larger Gondwanan landmass, Indo-Madagascar separated from other landmasses ~115 Ma, with complete isolation occurring ~88 Ma [2]. The ancestors of most of the island’s living groups appear to have arrived after the island was already isolated, possibly through rare “rafting” events [3].

Subsequent diversification and multiple devastating extinction events have also played a major role, as well as substantial climatic changes that affected both the island’s marine and terrestrial fauna. These include the Paleocene/Eocene thermal maximum (~55.8 Ma), the extreme lowering of temperature during the Grande Coupure at the Eocene/Oligocene boundary, and the shift to “modern” ocean circulation patterns during the mid-Miocene [4].

Madagascar’s living sharks and rays are thought to exceed 100 modern species [5]. While Madagascar is known for producing outstanding fossils, the record of selachians is relatively poor being restricted to the Triassic [6], and Upper Cretaceous [7–9], Eocene [10,11] and Miocene [9,12].

The only other study of Madagascar’s Miocene selachians recorded “*Carcharodon*” *megalodon*, *Odontaspis*, “*Carcharias*”, *Galeocerdo*, “*Sphyrna*”, *Hemipristis* and *Myliobatis* from Nosy Makamby [12]. We report here the first comprehensive fossil selachian assemblage from the Miocene of Madagascar, likely from the same layer, consisting of isolated teeth from ten taxonomic groups representing at least 12 species, six of which are newly described from Madagascar: *Galeocerdo*, *Rhizoprionodon*, *Sphyrna*, *Squatina*, *Rostroraja* and *Himantura*. We include comparisons with other contemporaneous faunas and explore environmental indicators and other associated taxa to help shed light on this region’s paleoenvironment.

## Geology and Age

**Fig 1:**
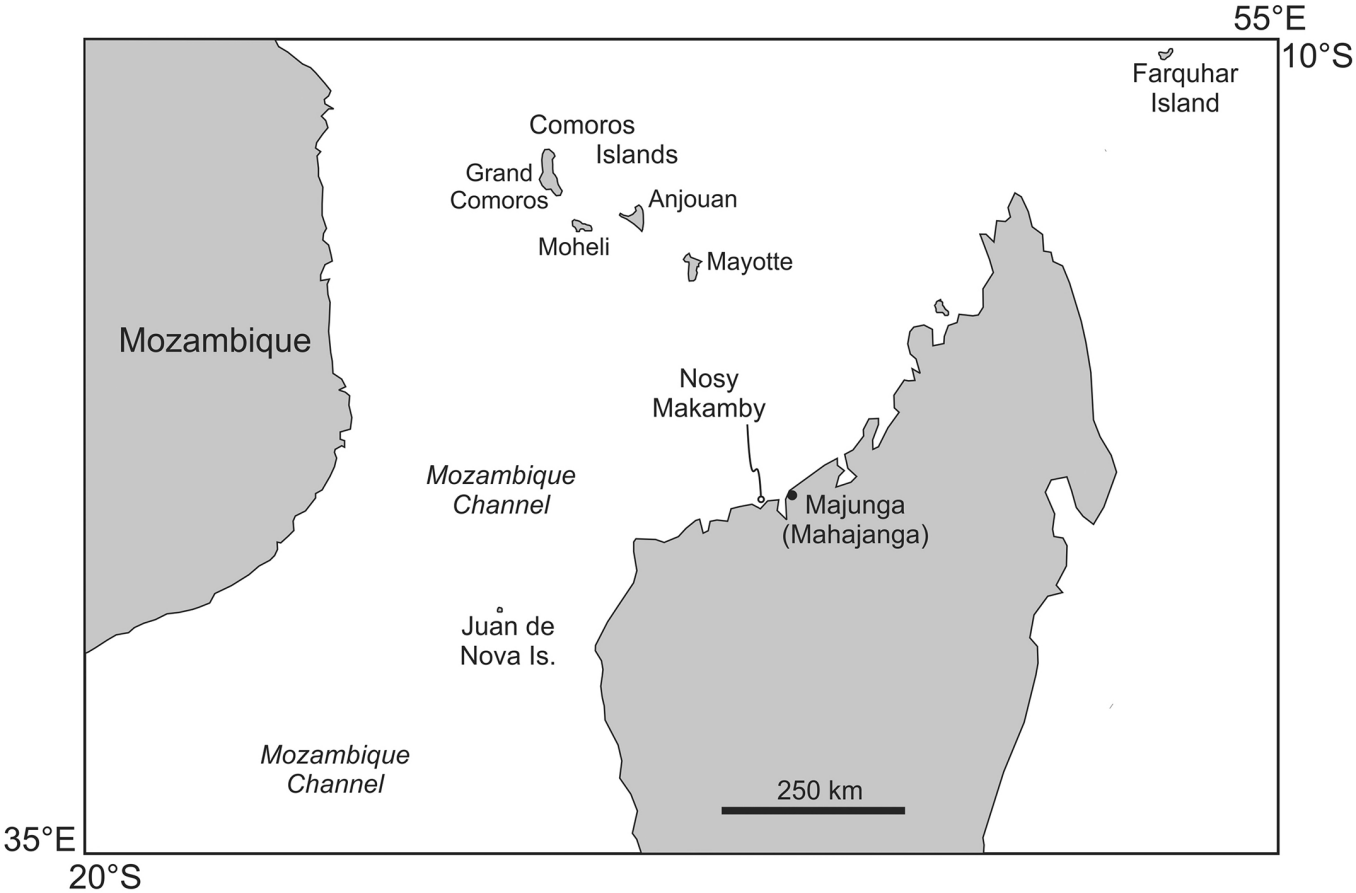
Regional map showing location of the study site, the island of Nosy Makamby, northwestern Madagascar. Also indicated is the port city of Mahajanga.

**Fig 2:**
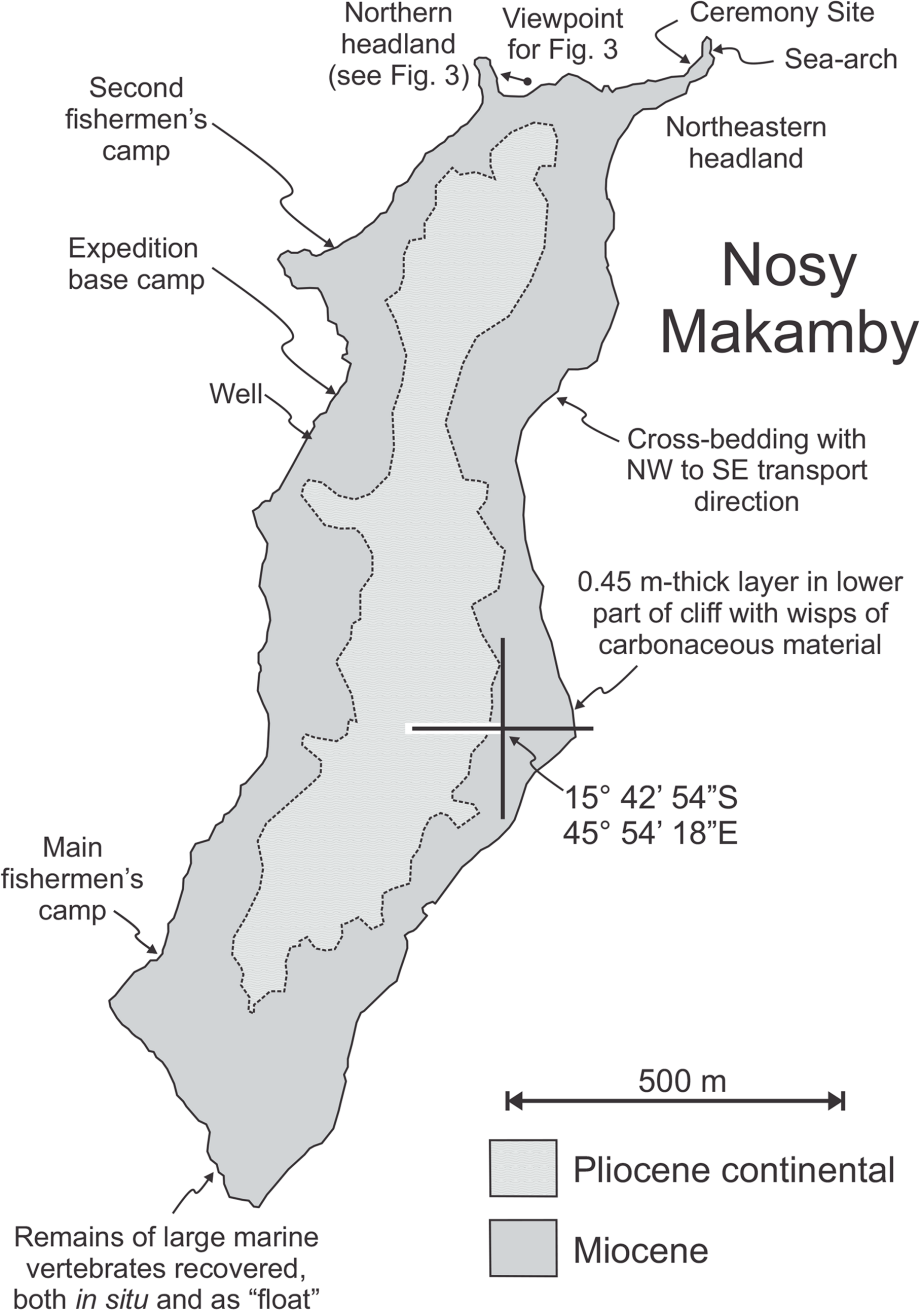
Simplified map of Nosy Makamby, northwestern Madagascar. Within the Miocene, the oldest exposed levels occur midway down the eastern coast.

Nosy Makamby (= Mahakamby) is a small (~1.6 km x 0.4 km) island SSW-NNE aligned offshore at broad of the delta of Mahavavy River, in northwest Madagascar, approximately 50 km west along the coast from the regional capital of Mahajanga (Figs 1 and 2). Very little geological information has been reported from Nosy Makamby and surrounding areas [13,14]; the only comprehensive description of the island’s fossils is the result of reconnaissance work done in the early part of the last century [12]. Recent exploration has produced a diverse assemblage of both invertebrates and vertebrates from Nosy Makamby, including foraminiferans, bivalves, gastropods, crabs, echinoids, sharks, non-diagnostic reptiles (turtles and crocodylians), and sirenians [12,14–16].

**Fig 3:**
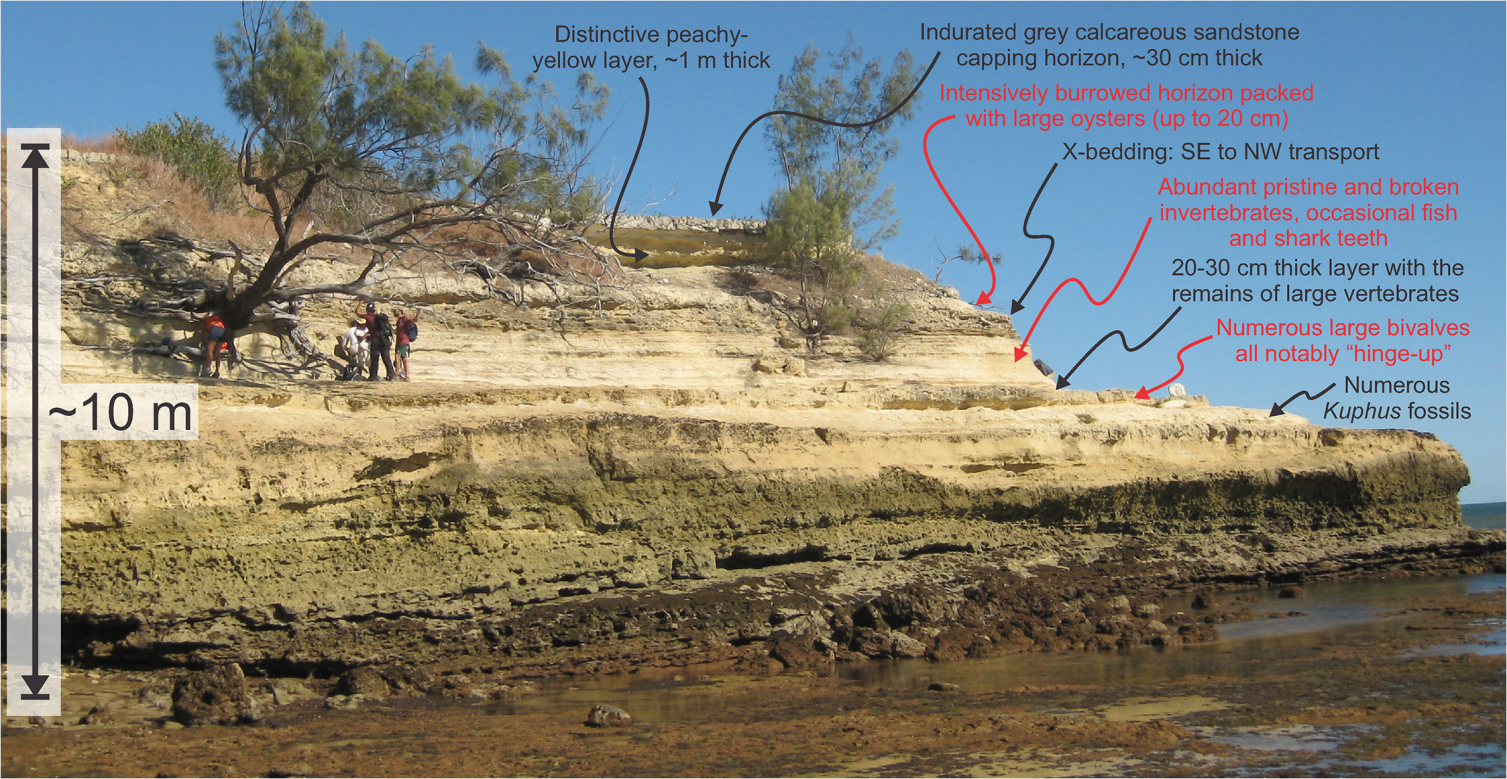
Stratigraphic summary figure for the northern headland of Nosy Makamby. The view is towards the south and represents the profile of the eastern side.

Nosy Makamby exposes one of the thickest and most complete sedimentary layers of marine Miocene in Madagascar, with lateral extensions of the succession in the regions of Cap Tanjona, Cap Sada, and Amparafaka to the west [12]. Two “formations” are exposed on the island (Fig 2)–a Miocene clastic unit, consisting mainly of medium to coarse sandstones that accumulated in a coastal plain/near-shore marine environment, and a Pliocene continental unit comprising red beds and quartz grits [12,14] (Fig 3). Geological sections on Nosy Makamby expose about 15 m of Miocene sediments. Interestingly, the thicknesses of the lithostratigraphic units mentioned in previous work are almost exactly an order of magnitude greater that the ones we observed, presumably this error occurred when Collignon and Cottereau published their manuscript [12]. A key marker bed is the informally designated “Ceremony Site Horizon” (FCSH), its “type-locality” being a 25-m-long by 10-m-wide platform on the north-eastern headland. Using this horizon together with a ~2.5 m-thick package of rocks immediately below that is rich in *Kuphus* tubes [17] makes the tracing of levels between exposures on the north of the island straightforward.

The sedimentary facies associated with the Miocene rocks are highly suggestive of a near-shore/estuarine/coastal plain depositional environment (probably not unlike the mouth of the present-day Betsiboka) possessing course sands containing significant amounts of pristine and abraded biogenic material and cross-bedding indicating transport directions in opposites directions (but without herring-bone cross-bedding). While the geologic section suggests a tidal, very shallow environment, there is a decided lack of plant debris in the sediments. The only level where such material was found occurs in 0.4-cm thick bed along the eastern coast, ~8 m below the FCSH (Fig 2). Clearly not much carbonaceous material found its way into these deposits, which might provide clues as to the nature of the vegetation and/or the climate system in the Mahajanga Basin during the Miocene.

Shark teeth come from the horizon captioned as “Abundant pristine and broken invertebrates, occasional fish and shark teeth” (Fig 3). While there is no specific locality information in Collignon and Cottreau [12] we assume that our fossils come from the same layer as described in their paper.

## Methods

Fossils were obtained through surface collection as well as both wet and dry screening. Residue from screening was broken down in the laboratory using acetic acid preparation techniques [18]. Residue was placed into a dilute (~5%) solution of acetic acid buffered with calcium orthophosphate. After each acid treatment, pieces were placed in water and rinsed thoroughly until completely free of acetic acid. Material disaggregated from the blocks was put through a 0.5 mm sieve and rinsed until clean. Samples were then air dried and sorted under a microscope. Photographs and standard measurements were taken, where appropriate, to aid in identification. Measurements were made with 500–172 Mitutoyo digital calipers to 0.1 mm. All specimens mentioned in this paper are deposited in the Laboratory of Paleontology and Biostratigraphy (Department of Paleontology and Biological Anthropology, UAP = Université d’Antananarivo, Antananarivo, Madagascar). All necessary permits were obtained from the Malagasy Ministry of Mines for the described study, which complied with all relevant regulations (001/2005; 002/2010;003/2011; 001/2013; 001/2014).

## Systematic Paleontology

For the classification of higher taxa as well as stratigraphic and geographic distribution see Cappetta (2012) [19]. The size of the specimens can be extrapolated from the plate and are given where appropriate.

Class Chondrichthyes Huxley, 1880

Subclass Elasmobranchii Bonaparte, 1838

Superorder Galeomorphii Compagno, 1973

Family otodontidae Glickman, 1964

Genus *Otodus* Agassiz, 1843 *sensu* Cappetta, 2012

*Otodus megalodon* Agassiz, 1835

Fig 4A

**Fig 4:**
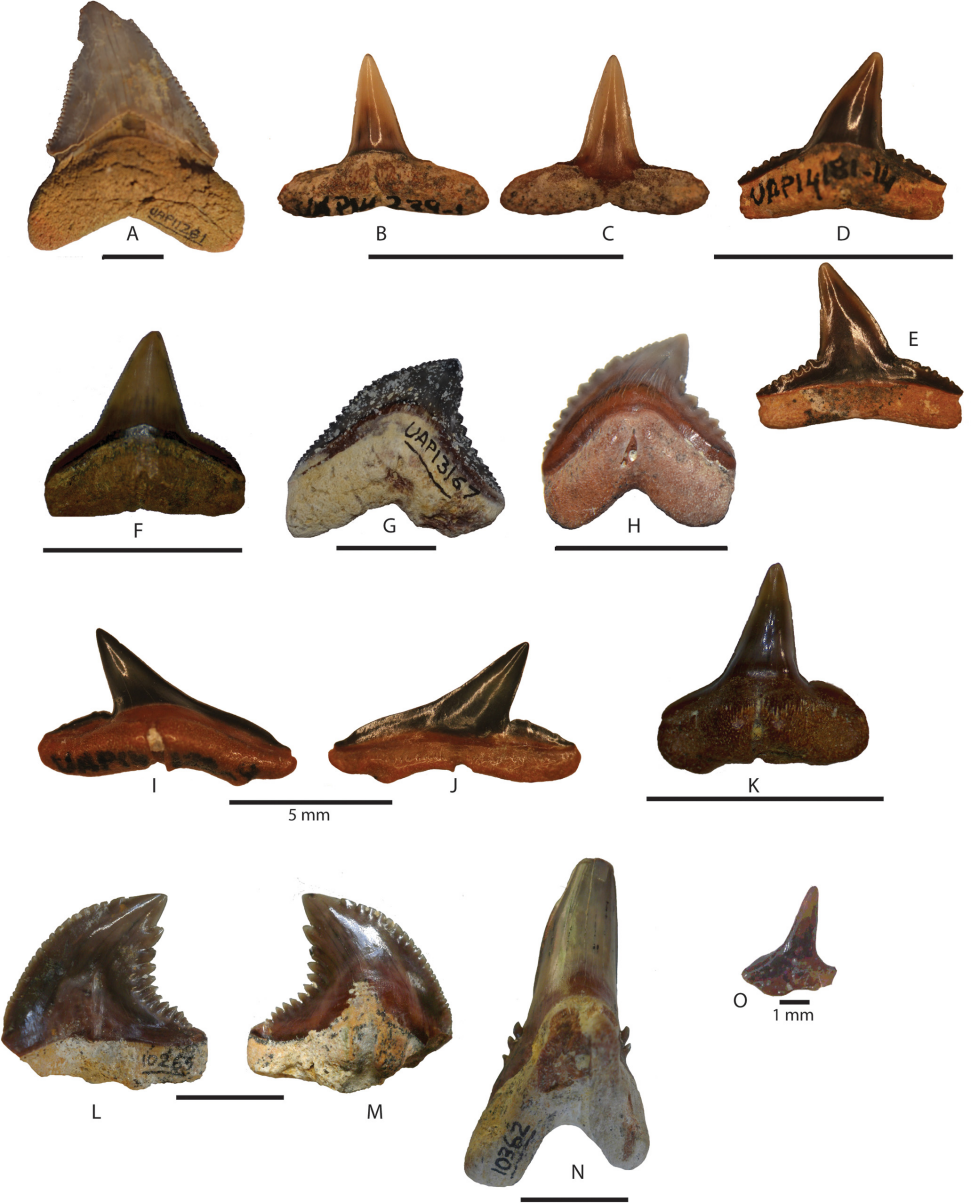
Miocene shark teeth from northwestern Madagascar. A, *Otodus megalodon* UAP-11.281; B-E, *Carcharhinus priscus*, B, UAP-14.239–1, lower tooth in labial view; C, UAP-14.239–1, lower tooth in lingual view; D, UAP-14.181–14, upper tooth in labial view; E, UAP-14.181–14, upper tooth in labial view; F, *Carcharhinus* sp. UAP-13.159; G-H, *Galeocerdo mayumbensis* UAP-13.167, UAP-13.172; I-J, *Rhizoprionodon ficheuri* UAP-14.122–9 I, labial view; J, lingual view; K, *Sphyrna* sp. UAP-13142; L-N *Hemipristis serra* UAP-10.362; O, *Squatina* UAP-10.505. Scale bar equals 10 mm, unless specified in the Figure.

### Synonomy and selected references

See [19] for both a description and a discussion of the use of the genus *Otodus* for this lineage.

### Material

One tooth (UAP-11.281).

One right upper anterior tooth from a juvenile individual 41.0 mm high and 34.5 mm wide at the base.

### Discussion

This species of shark (often referred to as a "Megatooth shark") is one of the more common and most iconic Cenozoic fossil vertebrates. It is frequently and erroneously, referred to as "*Carcharodon megalodon*" implying a close relationship with the great white shark. It was also referred to the genera *Procarcharodon* Casier, 1960 and *Carcharocles* Jordan and Hannibal, 1923. Ward and Bonavia [20] briefly discussed the taxonomy, ontogeny and species concepts. In the Miocene, teeth of juvenile individuals of *C*. *megalodon* bear lateral cusps which progressively diminish in size with age, the adults having completely lost them [20]. This particular morphotype is usually referred to as *O*. *chubutensis* [21]. Collignon and Cottreau [12] list three teeth of *Otodus megalodon* (as *Carcharodon megalodon*) from Makamby. They liken one of them to an early Miocene tooth, bearing small rounded, almost vestigial lateral cusps, figured by Priem (p. 122, pl. III, Fig 4 [22]) from the south of France which Priem referred to as *O*. *megalodon* var. *productus* (Agassiz 1843). As our only specimen has damage to both the medial and distal cutting edge at the crown base, it is impossible to say whether it would have corresponded to this morphotype.

Order Carcharhiniformes Compagno, 1973

Family Carcharhinidae Jordan & Evermann, 1896

Genus *Carcharhinus* Blainville, 1816

### Description

See Cappetta (p. 301, Fig 285) [19] for images of upper and lower teeth of an assortment of Recent species. Teeth of *Carcharhinus* generally exhibit dignathic and gradient heterodonty. Upper teeth are broadly triangular, usually slightly distally directed and serrated. The labial face is flat and does not possess a basal bulge; lingual face is slightly convex. Lower teeth are generally an inverted "T" shape with a narrower more upright crown and wide root and usually lightly serrated. Cusps are narrower and the labial face of the crown occasionally overhangs the root [19].

*Carcharhinus priscus* Agassiz, 1843

(Fig 4B–4E)

### Synonomy and selected references

See Reinecke *et al*. [23] and Bor *et al*. [24].

### Material

Ninety three teeth (UAP-05.378, UAP-10.219, UAP-10.269, UAP-10.272, UAP-10.311, UAP-10.340, UAP-10.343, UAP-10.346, UAP-10.364, UAP-10.367, UAP-10.369, UAP-10.370, UAP-10.399, UAP-10.418, UAP10445, UAP-10.449, UAP10450, UAP-10.425, UAP-10.451, UAP-10.459, UAP-10.501, UAP-10.504, UAP-11.160, UAP-11.164, UAP-11.166, UAP-11.170, UAP-11.174, UAP-11.192, UAP-11.233, UAP-11.234, UAP-11.240, UAP-11.167, UAP-11.178, UAP-11.81, UAP-11.193, UAP-11.199, UAP-11.215, UAP-11.232, UAP-11.271, UAP-11.286, UAP-11.287, UAP-11.352, UAP-13.010–1, UAP-13.103, UAP-13.130, UAP-13.127, UAP-13.129, UAP-13.132, UAP-13.134, UAP-13.139, UAP-13.141, UAP-13.148, UAP-13.150, UAP-13.157, UAP-13.158, UAP-13.160, UAP-13.161, UAP-13.010–1, UAP-13.110, UAP-13.119, UAP-13.127, UAP-13.129, UAP-13.132, UAP-13.141, UAP-13.145, UAP-13.146, UAP-13.148, UAP-13.150, UAP-13.157, UAP-13.158, UAP-13.160, UAP-13.161, UAP-14.122–3, UAP-14.122-, UAP-14.122–5, UAP-14.122–8, UAP-14.128–1, UAP-14.128–2, UAP-14.129–3, UAP-14.181–3, UAP-14.181, UAP-14.181–10, UAP-14.181–11, UAP-14.181–12, UAP-14.181–14, UAP-14.181–19, UAP-14.202–2, UAP-14.215–2, UAP-14.215–4, UAP-14.215–5, UAP-14.239–1, UAP-14.239–6).

### Description

See Reinecke *et al*. [23] and Bor *et al*. [24] for an extensive review of *C*. *priscus*. Upper teeth of *C*. *priscus* are characterized by having an unserrated or very lightly serrated cusps and moderately serrated crown shoulders. The crown is triangular and has uniform serrations along the entire border. Teeth are straight in labial view and the lingual surface is slightly convex. Root has a central foramen. Lower teeth have a narrower cusp well separated from the heels.

### Discussion

The teeth from Nosy Makamby correspond quite closely to some of Agassiz's types from the early Miocene of Malta (Tome III, p. 235,235, pl. 26a, Figs 44, 47, 48) and also those figured by Reinecke *et al*. p. 64, pls 71–76 [23]. *Carcharhinus priscus* was recorded by Collignon and Cottreau [12] from Makamby under the name *Sphyrna prisca* Agassiz. It is very likely that their records of "*Carcharias* (*Prionodon*)" and "*Carcharias* (*Aprionodon*)" were also based on teeth of *C*. *priscus*, the latter being lower teeth. The genus "*Carcharias*" was used for teeth we now refer to *Carcharhinus* currently well into the 20th century and was used by Priem [22] in a paper on Neogene shark teeth from southwest France. It is likely that this publication was used by Collignon and Cottreau [12] for their identifications. *C*. *priscus* is the most common species of *Carcharhinus* in the European Miocene and probably gave rise to the Recent species *C*. *brachyurus* Günther, 1870, *C*. *limbatus* Valenciennes, 1839, *C*. *perezii* Poey, 1876, among others.

In contrast to the somewhat restricted NW European assemblage, Purdy *et al*. [25] recorded a diverse *Carcharhinus* assemblage from the early Miocene Pungo River Marl, of Lee Creek, Aurora, North Carolina which they referred to Recent species.

Considering the variability observed in the *C*. *priscus* dentitions from NW Europe [23] and the conservative nature of teeth in some radiating shark lineages, one must consider the likelihood that *C*. *priscus* represents a species group rather than a discrete species.

*Carcharhinus* sp.

(Fig 4F)

### Material

Five teeth (UAP-13.159, UAP-11.134, UAP-11.243, UAP-11.351, UAP-13.159).

### Description

The tooth described (UAP-13.159) is small; 10.5 mm wide and 9.8 mm high. It has a low, finely serrated triangular crown flanked by evenly serrated lateral shoulders. The root is deep on the lingual aspect, and the labial surface bears a slight apically directed furrow.

### Discussion

The combination of fine, even serrations and a broad triangular shape with convex shoulders is not seen in any extant species of *Carcharhinus*. It most closely resembles upper teeth of the *obscurus—leucas- amboinensis—galapagensis—longimanus* group of *Carcharhinus* species. Broad triangular teeth appear in the late Eocene of northern and north-western Africa, relatively early in the *Carcharhinus* fossil record (Adnet *et al*., 2010, p. 864, Fig 3G [26]; Underwood *et al*., p. 54, Fig 4N [27]).

It is very similar in appearance to one of the syntypes of *Carcharias (Prionodon) similis* Probst, 1878 (Fig 12), refigured by Reinecke *et al*. (p. 77, text Fig 26 a-c) [23] and who regarded it as a median tooth of *Galeocerdo aduncus* Agassiz, 1835. The similarity is compelling. However, the Malagasy teeth differ in having a very low mesial protuberance, as opposed to the pronounced protuberance in Probst's specimen.

Genus *Galeocerdo* Müller & Henle, 1837

*Galeocerdo mayumbensis* Dartevelle & Casier, 1943

(Fig 4G–4H)

### Synonomy

1943 *Galeocerdo mayumbensis* sp. nov. Dartevelle & Casier, p. 153, pl. 12, Figs 22–30 [24].

1999 *Galeocerdo casei* sp. nov. Müller, p. 50 PL 11. 1–4. [28].

2011 *Galeocerdo mayumbensis* (name and figures only) Rathbone & Rathbone, p. 205 [29].

2015 *Galeocerdo mayumbensis* Argyriou *et al*. [30].

### Material

Twenty three teeth (UAP-05.377, UAP-10.208, UAP-10.267, UAP-10.270, UAP-10.308, UAP-10.310, UAP-10.363, UAP-10.499, UAP-11.082, UAP-11.149, UAP-13167, UAP-13172, UAP-11099, UAP-11187, UAP-11200, UAP-11279, UAP-11280, UAP-11306, UAP-13162, UAP-14128-5, UAP-14131, UAP-14143-1, UAP-14161-1).

### Description

See Cappetta (p. 298) [19] for a basic description of *Galeocerdo* teeth. Teeth of *G*. *mayumbensis* have a tall crown with a distally directed cusp with fine serrations. The mesial cutting edge is evenly convex while the distal heel is straight or slightly concave. Both are coarsely serrated; the larger, more apical serrae are themselves serrated. The distal notch between the cusp and the distal heel is reduced when compared with all other species of *Galeocerdo* [31].

### Discussion

The teeth of *G*. *mayumbensis* most closely resemble those of *G*. *eaglesomei* White, 1955 from the mid and late Eocene. They differ from *G*. *eaglesomei* in exhibiting less monognathic heterodonty and from *G*. *eaglesomei* and the middle Eocene species *G*. *latidens* in having compound rather than simple serrae on the mesial cutting edge and the distal heel. Teeth of the late Miocene to Recent species, *G*. *cuvier* Péron & Lesueur, 1822 differ from those of *G*. *mayumbensis* by being lower cusped but more robust with a more pronounced mesial cutting edge and curbed distal heel. In *G*. *mayumbensis* the distal heel is straight, the distal notch much less developed, and the root more arched.

The most common Miocene species of *Galeocerdo*, *G*. *aduncus* Agassiz, 1835, differs from *G*. *mayumbensis* by being smaller and having a less sigmoid crown and a more convex (curved) mesial cutting edge [30]. It displays strong monognathic, dignathic and possibly gynandric heterodonty, characters not present in teeth of the other species of *Galeocerdo*. Ward and Bonavia [20] synonymized *Galeocerdo contortus* Gibbes, 1849 and *Galeocerdo aduncus* and referred them to *Physogaleus*. This revision was rejected by Reinecke *et al*. (p. 79) [23] a view accepted here. However, as the dentition of *G*. *aduncus* lies between that of *Physogaleus* and *Galeocerdo* we feel the species *G*. *aduncus* would be better accommodated in a separate genus.

Teeth of *G*. *mayumbensis* have only been figured in scientific literature from the early Miocene of Cabinda and Bololo, Angola [32], from the eastern USA by Müller 1999 [28] and from the early Miocene of Libya by Argyriou *et al*. [30]. This species is well known to fossil collectors in Florida and a number are figured in a popular sharks' tooth identification guide [29]. No stratigraphic information was included, however it does occur in the phosphorite pebble beds in the mid to late Miocene Bone Valley Member of Hawthorn Group exposed off shore at Venice Beach, Florida (David J. Ward, personal observation).

Genus *Rhizoprionodon* Whitley, 1929

*Rhizoprionodon ficheuri* (Joleaud, 1912)

(Fig 4I and 4J)

### Material

Fifty two teeth (UAP-10.344, UAP-10.371, UAP-10.372, UAP-10.375, UAP-10.444, UAP-10.446, UAP-10.452, UAP-10.455, UAP-10.461, UAP-11.025, UAP-11.266, UAP-11.168, UAP-11.183, UAP-11.195, UAP-11.196, UAP-11.197, UAP-11.198, UAP-11.206, UAP-11.239, UAP-11.258, UAP-11.263, UAP-11.265, UAP-11.266, UAP-11.284, UAP-11.337, UAP-13.100, UAP-13.103, UAP-13.106, UAP-13.109, UAP-13.125, UAP-13.133, UAP-13.134, UAP-13.136, UAP-13.137, UAP-13.138, UAP-13.139, UAP-13.149, UAP-13.152, UAP-13.163, UAP-14.122–1, UAP-14.122–7, UAP-14.122–9, UAP-14.128–6, UAP-14.129–2, UAP-14.161–2, UAP-14.161–3, UAP-14.181–17, UAP-14.202–1, UAP-14.215–3, UAP-14.215–6, UAP-14.215–7, UAP-14.215–9).

### Description

Small wide teeth comprising distally directed crown and single distal cusplet which in lower teeth may be almost separate from the crown. This is more marked in male teeth.

### Discussion

Teeth of Recent species of *Rhizoprionodon*, *Loxodon* and *Scoliodon*, as well as those of some juvenile hammerheads (*Sphyrna*) exhibit a very similar morphology and are difficult to separate. The tooth figured (Fig 4I and 4J) is a wide lower lateral tooth, a shape more typical of *Rhizoprionodon*.

Family SPHYRNIDAE Gill, 1872

Genus *Sphyrna* Rafinesque, 1810

*Sphyrna* sp.

(Fig 4K)

### Material

Sixteen teeth (UAP-10.287, UAP-10.453, UAP-10.458, UAP-11.290, UAP-11.232, UAP-11.199, UAP-13.053–2, UAP-13.120, UAP-13.140, UAP-13.142, UAP-13.144, UAP-13.156, UAP-14.181–7, UAP-14.181–13, UAP-14.181–18, UAP-14.239–2).

### Description

UAP-13.142: Height = 7.3 mm, width = 8.1 mm, thickness = 2.4 mm.

Small triangular teeth with a single distally directed crown and low distal blade, both lacking serrae. Root short with central foramen. Crown with smooth edges, lacking crenellations and basal ledge on the labial aspect. The lingual root bears a well-defined notch at level of crown base.

### Discussion

While the teeth figured fall within the range of variation of the fossil species *Sphyrna integra* Probst, 1878 (figured by Reinecke *et al*.) [23] the sample size is too small for a confident identification. Purdy *et al*. [25] referred the teeth of hammerhead sharks from the Miocene and early Pliocene of Lee Creek Mine to three extant species: *S*. *lewini* Griffith & Smith, 1834, *S*. cf. *S*. *media* Springer, 1940 and *S*. *zygaena* Linnaeus, 1758; the latter they regarded as a senior synonym of *S*. *laevissima* Cope, 1867.

Family Hemigaleidae Hasse, 1879

Genus *Hemipristis* Agassiz, 1835

*Hemipristis serra* Agassiz, 1835

(Fig 4L–4N)

### Synonomy and selected references

See Cappetta [19] and Bor *et al*. [24].

### Material

Thirteen teeth (UAP-10.263, UAP-10.265, UAP-10.266, UAP-10.309, UAP-10.497, UAP-10.454, UAP-10.362, UAP-10.398, UAP-10.414, UAP-11.180, UAP-11.212, UAP-14.196, UAP-14.239–3).

### Description

A large well known species with marked gradient monognathic and dignathic heterodonty [19]. Upper teeth possess a triangular, high and thin crown that is bent at the rear [19]. Mesial cutting edge has well marked serrations that increase in size towards the apex, but do not reach the tip. The distal cutting edge has larger and less pointed serrations. Root is high and has a prominent lingual protuberance with a clear groove possessing one to several foramina. Lower teeth have a different morphology, with anterior teeth being high and sharp and lateral teeth possessing asymmetrical teeth with posteriorly bent cusps.

### Discussion

Throughout the Miocene and early Pliocene *H*. *serra* is a cosmopolitan species, occurring more commonly in warmer waters. The Malagasy specimens are smaller than those from the Miocene and Pliocene of North Carolina [25] and so may be from juvenile individuals.

Order Squatiniformes De Buen, 1926

Family Squatinidae Bonaparte, 1838

Genus *Squatina* Duméril, 1806

*Squatina* sp.

(Fig 4O)

### Referred Material

One isolated tooth (UAP-10.505).

### Description

UAP-10.505: height = 3.4 mm, width = 2.8 mm, thickness = 1.0 mm.

Small tooth with flattened root base. Crown upright, inclined disto-lingually with blade-like shoulders mesial and distal to the main crown. Sharp cusp in the anterior files. The basal face of the root in lateral teeth is flat.

### Discussion

Teeth of different *Squatina* species show very little variation and are difficult, if not impossible, to separate. See Bor *et al*. [24] and Ward and Bonavia [20] for further discussion. Bor *et al*. use the name *S*. *subserrata* Münster 1846 originally described from the Vienna Basin, for teeth that they figure from the Miocene of the Netherlands. However, considering the distances involved, we feel that open nomenclature is more appropriate.

Order Rajiformes Berg, 1937

Family Rajidae Blainville, 1816

Genus Rostroraja Hulley, 1972

*Rostroraja olisiponensis* Jonet, 1968

(Fig 5A)

**Fig 5:**
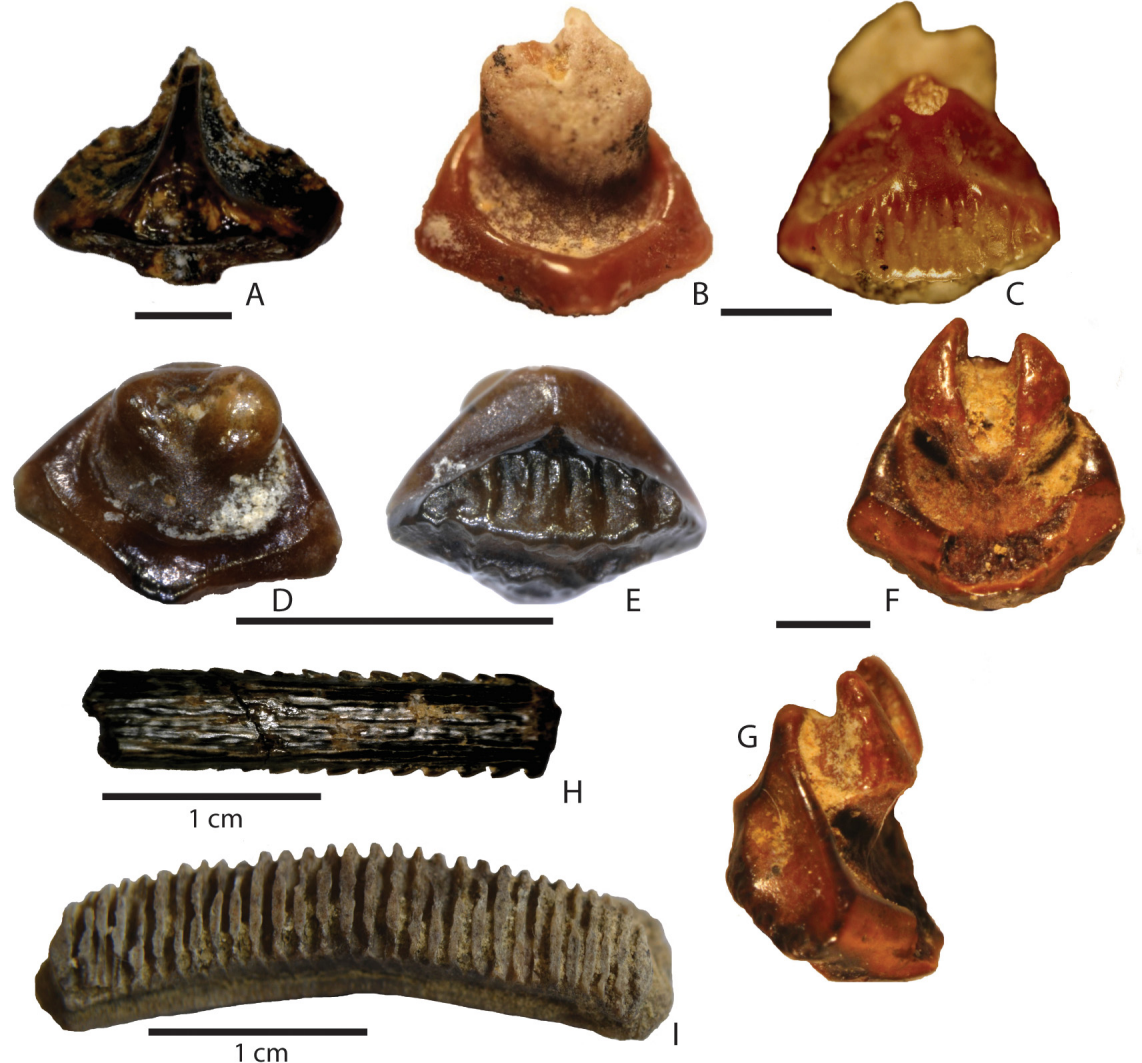
Miocene batoid teeth from northwestern Madagascar. A, *Rostroraja olisiponensis* UAP-13.016 (12); B-G, *Himantura*, B-C, UAP-11.311; D-E, UAP-13050; F-G, UAP-14.181; H-I, Myliobatidae indet. UAP-13.035, UAP-14.064. Scale bar equals 1 mm, unless specified in the Figure.

### Material

One isolated tooth (UAP-13.016).

### Description

Height = 3.8 mm, width = 2.9 mm, thickness = 2.5 mm.

Rajid tooth with a laterally expanded ovoid crown from which protrudes a lingually inclined conical cusp. Root is raised and broadly expanded mesial-distally.

### Discussion

This single tooth corresponds quite well with the type material [33] and those figured by Bor *et al*. [24]. This species displays a degree of ontogenetic heterodonty with relatively taller cusps present in larger teeth [33]. Bor *et al*. [24] suggest that this species may be ancestral to the extant white skate *Rostroraja alba* Lacépède, 1803, which occurs off the coast of east Africa, in the eastern Atlantic from Ireland and England southward round the Cape (South Africa) to central Mozambique [34].

Order Myliobatiformes Compagno, 1973

Family dasyatidae.Jordan, 1888

Genus *Himantura* Müller & Henle, 1837

(Fig 5B–5G)

### Material

Six isolated teeth (UAP-13.050 [lot of 6], UAP-13.050 [lot of 10], UAP-13.050 [lot of 20], UAP-13.050, UAP-14.181–1, UAP-14.215–8).

### Description

UAP-13.050 [lot of 6]: Average height = 1.9 mm, width = 1.6 mm, thickness = 0.9 mm. The labial crown is evenly pitted and slightly concave. There is a prominent, coarsely ridged transverse ridge. The lingual crown is smooth with a well-developed median lingual ridge, the base of which is developed, in presumed male specimens, into a small cusp. The root lobes are lingually placed, basally flat and separated by a broad furrow which bears a single foramina. In basal view, the labial visor is relatively broad with a narrow lingual visor.

### Discussion

Cappetta [19] comments that many pre-Miocene teeth referred to *Dasyatis*, may well be representatives of other genera including *Himantura*. The Nosy Makamby teeth correspond well with those of the Recent species *Himantura uarnak* Forsskål 1775 figured by Cappetta (2012, Fig 411) [19], which currently inhabits the coast of Madagascar. *Himantura* has been recorded from the late Miocene Baripada Beds in India (as *Dasyatis menoni* [35]) and as *Himantura* sp. from the Pliocene of Italy [36].

Teeth of small-toothed rays are rarely reported unless the locality and sediment is amenable to bulk sampling techniques and thus they are usually underrepresented in faunal lists. It is very likely that many more species, as well as more specimens of groups reported here, will be discovered with further sampling.

Family Myliobatidae Bonaparte, 1838

Myliobatidae indet.

(Fig 5H–5I)

### Material

Eight isolated teeth (UAP-13.013, UAP-13.035, UAP-13.060, UAP-13.071, UAP-14.133, UAP-14.166, UAP-14.215–1, UAP-14.064).

### Description

Isolated chevrons, occlusal face flat, root polyaulacorhizoid with lingually displaced lobes and grooves.

### Discussion

Unless the dentition is partially or wholly complete, myliobatid teeth are generally referred to "*Myliobatis* sp", where a more accurate determination would usually be "Myliobatidae indet." Isolated and incomplete median teeth of *Myliobatis*, *Rhinoptera*, *Aetomylaeus*, *Aetobatus* and *Pteromylaeus* are similar but can be separated by their general proportions, spacing and degree of lingual offset of the root lobes. In the case of *Rhinoptera*, there is very little lingual displacement in the root lobes, whereas in *Aetobatus* it is quite marked [19]. It is likely that the three figured teeth are of at least two different genera as their general proportions and lingual root offset differ significantly.

## Results and Discussion

### Comparisons with other contemporaneous faunas

The shark and ray teeth collected from the sections on Nosy Makamby show a degree of Recent weathering but no indication of having been reworked. Thus it is reasonable to assume that they are the same age as the surrounding sediment. Most species have ranges that span the Miocene, and therefore offer little indication as to the specific age of the deposit. However, the presence of *Carcharhinus priscus* and *Galeocerdo mayumbensis* would suggest an early to middle Miocene age. The Miocene fauna described by Priem [37] from Chandane, Mozambique, contains a number of late Eocene species (*Carcharhinus frequens* Dames, *Galeocerdo latidens* Agassiz etc.), suggesting that the assemblage is of mixed age and not useful for comparison.

The shark fauna from the Chesapeake Bay is relatively well known [38] but for taphonomic reasons the teeth of rays are particularly rare. Both sharks and rays are relatively abundant in the early Miocene of North Carolina, USA [25] but the smaller sharks and rays are poorly known.

Perhaps the best fossil elasmobranch assemblage to compare with that from Nosy Makamby is that described from the late Miocene Baripada Beds in India [35]. These were originally thought to be early Miocene [39] but are currently thought to be late Miocene based on the occurrence of a short-ranged fossil suid [35]. This fauna, which contains the ubiquitous Miocene elements like *Hemipristis*, *Carcharhinus* and *Galeocerdo*, also contains a number of smaller species which, with a taxonomic review, may be comparable with those from the Madagascan Miocene.

### Environment

All shark genera identified occupy the neritic littoral zone, with the majority preferring tropical climates. The apparent absence of sand and mako sharks (*Carcharias* and *Isurus*) is unusual as their teeth are usually abundant in inshore Miocene deposits [23][25].

The association of sirenian fossils from the same deposits at Nosy Makamby also supports a nearshore marine, protected and calm environment that possessed sufficiently clear water and low depth. Foraminifera further support this paleoenvironmental interpretation, with groups recovered dominated by miliolids, especially *Quinqueloculina* [17]. This indicates an inner shelf deposit in a coastal environment, and warm temperature characteristic of a tropical area similar to that reconstructed for the selachian genera. The presence of the invertebrate species *Concavus concavus* Bronn 1831, also suggests that the medium was continuously subjected to the influence of the tide, which may explain the thick lumachellic deposits. This appears to be characteristic of other Miocene formations near the Mozambique Channel (e.g., Tanzania) [40].

## Conclusions

Recent fieldwork on the island of Nosy Makamby, northwestern Madagascar, has produced the first comprehensive description of the island’s Miocene selachians. Of the ten taxonomic groups identified, *Otodus*, *Carcharhinus*, *Galeocerdo*, *Rhizoprionodon*, *Sphyrna*, *Hemipristis*, *Squatina*, *Rostroraja*, *Himantura* and Myliobatidae, six are newly described from Madagascar (*Galeocerdo*, *Rhizoprionodon*, *Sphyrna*, *Squatina*, *Rostroraja* and *Himantura*). This analysis of selachian remains combined with lithological data, and the further presence of sirenian and *Concavus concavus* fossils supports the age of early to mid Miocene, and suggests that this region was characterized as tropical shallow-water marine. Future work is needed to better understand the precise age, biostratigraphy and paleoenvironment of this unique island.
